# Khat Use Is Associated with Impaired Working Memory and Cognitive Flexibility

**DOI:** 10.1371/journal.pone.0020602

**Published:** 2011-06-15

**Authors:** Lorenza S. Colzato, Manuel J. Ruiz, Wery P. M. van den Wildenberg, Bernhard Hommel

**Affiliations:** 1 Institute for Psychological Research and Leiden Institute for Brain and Cognition, Leiden University, Leiden, The Netherlands; 2 Psychology Department, Amsterdam Center for the Study of Adaptive Control in Brain and Behaviour (Acacia), University of Amsterdam, Amsterdam, The Netherlands; Cuban Neuroscience Center, Cuba

## Abstract

**Rationale:**

Khat consumption has increased during the last decades in Eastern Africa and has become a global phenomenon spreading to ethnic communities in the rest of the world, such as The Netherlands, United Kingdom, Canada, and the United States. Very little is known, however, about the relation between khat use and cognitive control functions in khat users.

**Objective:**

We studied whether khat use is associated with changes in working memory (WM) and cognitive flexibility, two central cognitive control functions.

**Methods:**

Khat users and khat-free controls were matched in terms of sex, ethnicity, age, alcohol and cannabis consumption, and IQ (Raven's progressive matrices). Groups were tested on cognitive flexibility, as measured by a Global-Local task, and on WM using an N-back task.

**Result:**

Khat users performed significantly worse than controls on tasks tapping into cognitive flexibility as well as monitoring of information in WM.

**Conclusions:**

The present findings suggest that khat use impairs both cognitive flexibility and the updating of information in WM. The inability to monitor information in WM and to adjust behavior rapidly and flexibly may have repercussions for daily life activities.

## Introduction

The khat plant (*Catha Edulis*) is a flowering evergreen tree that grows at high altitudes. It is cultivated especially in East-Africa and the south west of the Arabian Peninsula, in countries such as Somalia, Kenya, Djibuti, Yemen, or Ethiopia. In those countries the chewing of khat is also very common; it is consumed as qat and kat in Yemen; eschat in Ethiopia; miraa, kijiti, gomba, mbachu or veve in Kenya; and as mairungi in Uganda.

Historically, khat leaves have been chewed since ancient times to alleviate fatigue, enhance work capacity, stay alert, reduce hunger, and to induce euphoria and enhanced self-esteem [1]; [2]. Khat has been appreciated for medical purposes too [3], and even for its aphrodisiac effects, but it is also used for recreational purposes [4]. It is habitually used in informal meetings (khat sessions) in which the participants are engaged in discussions and maintain social contact. During khat sessions, the leaves and the tender younger stalks of the plant are chewed slowly over several hours and they are kept in the side of the cheek until the mouth is filled with fresh leaves. The users then chew intermittently to release the active components and then spit out the residues [5].

The half life of khat is about 4 hours, depending on the amount of chewed khat. When the acute effects disappear, consumers experience feelings of depletion, insomnia, numbness, depression, lack of energy, and mental fatigue. Long-term, chronic (i.e. daily) use of khat is associated with increased blood pressure, development of gastrointestinal tract problems, cytotoxic effects on liver and kidneys, and keratotic lesions at the side of chewing [5].

Many authors have argued about the causal role of khat in exacerbating psychotic reactions. In psychotic patients, khat may aggravate thought disturbances (hallucinations and delusions), induce aggressive behavior, and create difficulties in treating these patients [6]; [7]. Regular users with a predisposition to psychotic symptoms, including schizotypal or schizoid traits and family disorders, also have an increased risk of khat-induced psychosis. The psychotic symptoms are abated rapidly when khat is withdrawn [8]; [9]. However, recently, Odenwald [10] challenged this assumption concluding that the causal relationship between general psychopathology and khat use remains unclear and that people with preexisting vulnerability should avoid using khat. Socio-economic and familiar problems may also arise in khat consumers [11]; [12]; [13]. Many men secure their daily portion of khat at the expense of vital needs, indicating dependence. Family life is harmed because of neglect, dissipation of family income, and inappropriate behaviour. In countries like Ethiopia or Kenya, khat-dependent individuals are the main group among those treated for drug problems [14].

The active ingredients of *Catha Edulis* are cathine (norpseudoephedrine) and cathinone (Benzoylethanamine). These alkaloids are similar in structure and pharmacological activity to amphetamines [15]. The acute effects of cathinone and cathine on neurotransmitters are basically comparable to amphetamines effects: both stimulate the CNS and suppress appetite. However, cathinone has a more rapid onset and a shorter half life than amphetamine. The two alkaloids act by increasing dopamine (DA), serotonin and noradrenaline [7]. For this reason khat is called a “natural amphetamine”. Even though the literature on the effect of *Catha Edulis* compounds on humans is scarce, khat is considered to increase blood pressure and heart rate, and is associated with euphoregenic and psychoestimulants effects [1].

Cathinone is probably the main contributor to the stimulant effect of khat. Cathinone is an unstable molecule that rapidly transformes into cathine. Cathine is a less powerful stimulant and the pharmacological conversion from cathinone to cathine causes the decrease of stimulating properties of khat leaves over time. Fresh leaves have a greater ratio of cathinone to cathine than dried ones [16]. Therefore, the fresh leaves have more psychoactive effects and a number of techniques are in use to slow down the degradation process (e.g., wrapping the khat in banana leaves). To provide consumers with fresh leaves, khat is delivered by air around the world, commonly no later than five days after been harvested. When the leaves are chewed, cathinone is absorbed through the buccal mucosa and the stomach. After absorption it is metabolically transformed into norephedrine [17]. The effects of oral administration of cathinone occur more rapidly than the effects of amphetamine; roughly 15 minutes as compared to 30 minutes in amphetamine. Cathinone increases levels of DA in the brain by acting on the cathecholaminergic synapses, delaying DA reuptake inactivation and/or enhancing DA release [18]; [15], in particular in the striatum [19]. However, it is important to note that the consumption of cathinone in pure form is not entirely comparable with chewing khat leaves.

Studies addressing the neurobiological mechanism underlying the use of khat are still missing as well as studies that have systematically investigated the long-term cognitive effects of chronic khat use. Nevertheless, given the similarity of khat and amphetamine in structure and pharmacological activity, it makes sense to assume that the long-term use of khat affects the same neurotransmitter and brain structures as the chronic use of amphetamine (see [20]). At a structural level, one may thus expect white matter abnormalities, lower cortical gray matter volume, and higher striatal volume. In particular, higher striatal volumes might reflect a compensation for toxicity in the dopamine-rich basal ganglia. At a functional level, in turn, chronic khat use is likely to be associated with reduced functioning of Dopamine D2 (DAD2) receptors in the striatum and dysfunctions in prefrontal cortex (PFC) and orbitofrontal cortex (OFC)—areas that have been shown to play major roles in cognitive control [21].

Interestingly, DA has a key role in WM processes [22]; [23] and in the ability to flexibly alter cognitive representations [24]. According to Moustafa et al. [25], the striatum serves as a gate to modulate when and when not to update information into PFC. Consistent with this idea, Siessmeier et al. [26] found that administering DA agents to healthy subjects leads to a correlation between DA uptake in the striatum and dorso-lateral PFC (DLPFC) BOLD activity, suggesting that the striatum might drive activity in the PFC. Moreover, a PET study showed that working memory capacity predicts dopamine synthesis capacity in the striatum [27]. Consistent with these findings, previous studies on chronic amphetamine users and mice provided evidence for impairment on WM due to amphetamine use [28]; [20]; [29]; [30]. Similarly, studies investigating patients with Parkinson's disease (PD), a neurodegenerative disorder characterized by severe DA depletion in the striatum, showed decrements for the flexible alteration of cognitive representations [24]. Along the same lines, there is evidence of decreasing mental flexibility due to amphetamine use [31].

Khat consumption in Eastern Africa has increased during the last decades and has become a global phenomenon spreading to ethnic communities in the rest of the world, such as in the Netherlands, United Kingdom, Canada, and the United States [14]. Amsterdam airport, where a large amount of khat arrives weekly, has become a European distribution point [32]; [13]. In the Netherlands, khat bundles are commonly sold in restaurants, grocery stores and smartshops, which makes this country a suitable platform to investigate the effects of the drug.

Surprisingly, only one study has systematically looked into cognitive impairments in khat users so far. Colzato et al. [33] reported that khat users exhibit impairments in the inhibition of overt manual responses assumed to rely on proper dopaminergic functioning [34]. The ability to inhibit unwanted thoughts and actions is commonly considered an important part of executive control, but it represents just one of a larger set of cognitive control functions. In an attempt to categorize the available concepts and measures in a coherent fashion, Miyake and colleagues have investigated the psychometric relationships between the tests and tasks that are commonly used to assess cognitive control [35]; [36]; [37]. Their findings suggest the existence of three major, separable control functions: the “inhibition” of unwanted responses, the “shifting” between tasks and mental sets (also called “flexibility”), and the “updating” (and monitoring of) working memory (WM) representations. Miyake et al. 's model has been previously used to investigate cognitive impairments among recreational users of cocaine and MDMA [38]; [39].

Given the link between khat use and impaired inhibitory control, the current study focused on the two remaining cognitive control functions; flexibility and updating. Importantly for the current study, the above mentioned links between DA and “updating” (and monitoring of) WM representations and mental flexibility on the one hand, and between DA and khat on the other, suggest that WM monitoring and mental flexibility are impaired in khat users. We tested both hypotheses by comparing khat users and matched khat-free controls in a task assessing the efficiency of monitoring information in WM and a task that taps into cognitive flexibility.

## Materials and Methods

### Participants

Forty young healthy adults (36 men and 4 women) were compensated for their participation. They constituted the two groups of 20 khat users and 20 khat-free controls. The sample was drawn from 50 adults in the Leiden and The Hague metropolitan area, who volunteered to participate in studies of behavioral pharmacology. Participants were recruited via ads posted on community bulletin boards and by word of mouth. Participants were selected via a phone interview. Based on the interview, we excluded 10 of the 50 potential participants because of current medication use.

We made sure that the users met the following criteria: (1) khat consumption by chewing route for a minimum of 1 year; (2) no clinically significant medical disease and (3) no use of medication. All khat users met more than four of the seven criteria that according to the American psychiatric Association DSM-IV and the World Health Organization (ICD-10) define addiction: tolerance, withdrawal, difficulty controlling the use, negative consequences for job, family and health, significant time or emotional energy spent in searching/consuming the drug, put off or neglected activities because of the use, and desire to cut down the use. None of the khat-free controls reported any history of past or current khat use.

Participants were asked to refrain from taking any psychoactive drugs for at least 24 hours before the test, not to consume alcohol on the night before the experimental session, and to have a normal night rest. Participant's compliance with the instruction was encouraged by taking a (not further analyzed) saliva sample test at the beginning of the session (cf. [38]; [40]).

The two groups were matched for ethnicity (100% African), age, sex, IQ, (measured by Raven's Standard progressive matrices (SPM); [41]) and alcohol and cannabis consumption. Even though khat was the preferred drug for users, some of them drink alcohol (7) on a weekly base and used cannabis (3) on a monthly base. Khat users and non-users reported to have never used LSD, MDMA, cocaine, amphetamine, barbiturates, ketamine, GHB or speed. Demographic and drug use information are provided in Table 1. Written informed consent was obtained from all participants after the nature of the study was explained to them. The protocol and the remuneration arrangements of 25 Euro were approved by the institutional review board (Leiden University, Institute for Psychological Research).

**Table 1:** Demographic characteristics and self-reported use of khat and other psychoactive drugs.

Sample	Khat users	Khat-free controls
N (M:F)^a^	20 (18:2)	20 (18:2)
Age (years)^a^	31.3 (6.5)	30.7 (5.8)
Raven IQ^a^	110 (3.3)	112 (3.0)
Khat exposure (years)**	10.5 (6.5)	0
Khat times in a week**	3.1 (1.8)	0
Bundles used (khat shrubs)**	3.0 (1.2)	0
Bundles used in one session	1.0 (1.9)	0
Hours chewing khat**	5.8 (1.7)	0
Monthly exposure (joints)^a^	2.0 (0.4)	2.1 (0.3)
Monthly drinks (units)^a^	8.4 (0.5)	6.4 (0.6)
Lifetime cocaine (grams)^a^	0	0
Lifetime amphetamines (grams)^a^	0	0
Lifetime ketamine (grams)^a^	0	0
Lifetime Speed (grams)^a^	0	0

Standard deviations are presented within parentheses.

*Raven IQ*: IQ measured by means of the Raven's Standard Progressive Matrices,

*Bundles used:* number of khat bundles consumed in a typical day/session

*Hours chewing khat*: amount of time the users spend chewing khat in a typical day/session

*Monthly drinks*: monthly number of standard alcoholic drinks

a Nonsignificant group difference

*Significant group difference, p<0.05

**Significant group difference, p<0.01

### Computerized tasks

The tasks used in this study have been previously employed to systematically investigate the neurotoxic effects of recreational MDMA use [30]; [39] and recreational use of cocaine [42]; [38]. Similar to these studies and following Miyake et al. [37], we defined cognitive flexibility as the ability to adapt and restructure cognitive representations in response to changing situational demands (cf., [43]). We used the N-back task (see, [44], for a recent review) to assess the operational component of WM and a task-switching design [45] to assess flexibility.

All participants were tested individually. Individual IQs were determined by means of a 30-min reasoning-based intelligence test (SPM). The SPM assesses the individual's ability to create perceptual relations and to reason by analogy independent of language and formal schooling; it is a standard, widely-used test to measure Spearman's g factor and of fluid intelligence in particular [41]. Participants provided a saliva sample, completed the SPM, and subsequently performed the behavioral tasks measuring cognitive flexibility and WM capacity. Participants were allowed to take a short break (maximal 10 minutes) between task blocks. The experiment was controlled by a PC attached to a 17-inch monitor with a refresh rate of 120 Hz. Participants were seated approximately 0.5 m from the screen.

#### N-back task (WM monitoring)

Participants performed two N-back tasks consisting of the sequential visual presentation (stimulus onset asynchrony 2000 ms; duration of presentation 1000 ms) of single letters (B, C, D, G, P, T, F, N, L). Participants had to press the left or right shift-key of the computer-keyboard when the target or the non-target appeared, respectively. Target definition differed with respect to the experimental condition. In the 1-back condition, targets were defined as stimuli within the sequence that were identical to the immediately preceding one. In the 2-back condition, participants had to respond if the presented letter matched the one that was presented two trials before. The 1-back, and 2-back tasks differ in their amount of memory load and demands on executive control for the processing of information within working memory. Each block consisted of four cycles of the same task.

#### Task switching (flexibility)

Participants responded to randomly presented rectangles or squares by pressing a left or right response button, respectively. The target stimuli contained a global dimension (i.e., the overal shape was either a rectangle or a square) and a local dimension (the overall shape consisted either of small squares or small rectangles). Three blocks of trials were administered, two training blocks in which the instruction (global or local) was constant across all trials, followed by the experimental block in which participants switched between the global and the local task. In one of the two training blocks, participants responded to the local figure, in the other block they responded to the global figure. The order of the training blocks was randomized across participants and each block consisted of 50 trials. In the third block consisting of 160 trials, participants alternated between predictable sequences of four “local” and four “global” trials). A cue indicated to which dimension (global or local) the participants should respond. Cues that related to the global (local) dimension consisted of a big (small) square, presented at one side of the target stimulus, and a big (small) rectangle, presented at the other side of the target stimulus. The color of cues and target was red. Both remained on the screen until a response was given or 2500 ms had passed. The time interval between presentation of the cue and of the target stimulus varied between 400 ms and 500 ms and the interval between responses and the next presentation of the cue varied between 900 ms and 1100 ms ms.

### Statistical analysis

We adopted a significance level of p<.05 for all statistical tests. Independent samples t-tests were used to analyze binary comparisons and ANOVAs otherwise.

T-tests were performed for Group analysis of age, sex, IQ and alcohol, and cannabis consumption and in the N-back task to assess differences between khat users and khat-free controls. For switching performance mean RTs and proportions of errors (PE) were analyzed by means of ANOVAs using Target level (global vs. local), the Congruency between the stimuli on the two levels (congruent vs. incongruent), and Task switch (i.e., same vs. different target level as in previous trial: task repetition vs. alternation) as within-participants factor and Group (khat users vs. khat-free controls) as between-participants factor. Spearman correlation coefficients were computed between the degree of exposure to khat and cognitive performance in order to test whether the magnitude of cognitive impairments is proportional to the amount of khat consumed. Effect magnitudes were assessed by calculating partial Eta squared (η2p) for repeated measures ANOVAs.

## Results

### Participants

No significant group differences were obtained for age, *t*(38) = 0.306, *p* = 0.761, intelligence, *t*(38) = −0.973, *p* = 0.337, alcohol consumption, *t*(38) = 0.478, *p* = 0.521, or cannabis consumption, *t*(38) = 0.169, *p* = 1.00. Table 1 shows drug-use profiles for the two groups.

### Tasks

The results per cognitive task are summarized below and in Table 2. The data of two male khat-users were excluded in both tasks because of their excessive error rates in the task-switching paradigm (PE>45%).

**Table 2:** Means responses latencies (in milliseconds), error rates (in percent), and standard deviations of all relevant measures for the N-back task and task switching.

Task	Khat users	Khat-free controls
N-BACK (WM monitoring/updating)		
1-back		
Reaction Times (ms)	494 (55)	504 (62)
Accuracy (%)	70 (17.5)*	91 (7.9)*
2-back		
Reaction Times (ms)	497 (68)	523 (50)
Accuracy (%)	62 (12.9)*	81 (11.4)*
TASK SWITCHING (flexibility)		
Repetition		
Reaction Times (ms)	494 (25)	455 (24)
Error Rates (%)	20.7 (1.6)	3.8 (1.5)
Alternation		
Reaction Times (ms)	581 (27)	492 (25)
Error Rates (%)	21.8 (1.7)	4.2 (1.6)
* Switch Costs*		
Reaction Times (ms)	87*	37*
Error Rates (%)	0.1	0.1

*Switch Costs Reaction Times:* difference in RT between alternation trials and repetition trials

*Switch Costs Error Rates:* difference in error rates between alternation trials and repetition trials

*Significant group difference, p<0.05 (referring to the interaction effect reported in the text)

#### Task switching

Analyses of Mean RT showed three reliable main effects (see Table 2). First, the effect of Task switch, F(1,36) = 38.30, p<.0001, MSE  = 7916.15, η^2^p  = 0.52, was due to that repeating the task allowed for faster responding than switching between target levels (475 vs. 537 ms). Second, the effect of Target level, F(1,36) = 23.52, p<.0001, MSE  = 8728.09, η^2^p  = 0.39, reflected the well-known global preference [46], that is, faster responses to globally than locally defined targets (480 vs. 532 ms). Third, the Congruency effect, F(1,36) = 7.46, p<.01, MSE  = 10881.70, η^2^p = 0.17, indicated interference from the non-target level, that is, faster responses if the stimulus at the currently irrelevant level was congruent (e.g., a global square shape consisting of local squares) with the present target than if that stimulus was incongruent (e.g., a global square consisting of local rectangles; 489 vs. 522 ms, respectively).

More important for present purposes, the size of the Switch effect varied by Group, *F*(1,36) = 5.68, *p<*.05, MSE  = 8073.45, *η* ^2^p  = 0.14: khat users showed more pronounced switching costs (i.e., a greater difference in RT between alternation trials and repetition trials) than khat-free controls. No other interaction was reliable.

Analyses of error rates revealed three reliable main effects. First, an effect of Group, F(1,36) = 73.91, p<.0001, MSE  = 3065.69, η2p  = 0.67: khat users committed significantly more errors than khat-free controls (21.3% vs. 4.0%). Second, the effect of Congruency, F(1,36) = 83.49, p<.0001, MSE  = 322.34, η2p  = 0.70, reflecting the interference of the irrelevant target level, as indicated by a smaller proportion of errors on congruent as compared to incongruent trials (3.3% vs. 22.1%). Third, the effect of Target level, F(1,36) = 5.17, p<.05, MSE  = 686.91, η2p  = 0.13, suggesting less errors to globally than locally defined targets (10.1% vs. 15.2%). No other effect was significant.

#### N-back task

Mean RTs and accuracy—with the latter commonly being the more reliable measure in this task—were submitted to independent t-tests, see Table 2 for means. As expected, khat users committed significantly more errors in both the 1-back, *t*(36)  = 4.72, *p* = 0.001, and 2-back conditions, *t*(36)  =  .75, *p* = 0.001, see Table 2. RTs revealed no significant group differences in the 1-back, *t*(36)  = 0.53, *p* = 0.59, and 2-back conditions, *t*(36)  = 1.37, *p* = 0.18.

### Correlations

To test whether the magnitude of cognitive impairments is proportional to the amount of khat consumed, we computed Pearson correlation coefficients between the individual life time khat exposure, hours chewing and number of bundles used in a khat session and switching costs, as well as accuracy in the n-back task. No significant correlations were obtained, probably due to the limited variability across users.

## Discussion

This study tested, for the first time, whether khat use is associated with a detectable selective impairment in cognitive flexibility and WM. As expected, khat users showed increased switching costs, suggesting that recreational use is associated with impaired cognitive flexibility. Performance in khat users differed from performance in non-users also with respect to WM updating (the executive component of WM). We attribute these deficits to the possibility that long-term use of cathinone, the active ingredient of khat, is associated with dysfunctions in PFC and a reduced DA level in the striatum—the neurotransmitter that plays a crucial role in cognitive flexibility and updating of WM [24]; [25]; [47]. Bearing in mind the similarity between cathinone and amphetamine, our results are also consistent with previous studies in humans showing impairments in WM [28]; [29]; [30]; [48] and cognitive flexibility [31] as consequences of long-term amphetamine and methamphetamine use.

Given that khat users committed significantly more errors also in the 1-back task, a condition that requires hardly any active maintenance of information, we suggest that khat use may be associated with impairment of the updating rather than the maintenance component of WM. Khat users may thus not necessarily process or store fewer items than khat-free controls but, rather, are less selective with regard to what they store [49]. In an N-back task, this would imply that khat users are less efficient in discriminating targets from non-targets, which means that non-targets are more likely to enter WM and interfere with target information.

Bearing in mind the similarity between cathinone and amphetamine, our results are also consistent with previous studies in humans showing impairments in WM [28]; [29]; [30]; [48] and cognitive flexibility [31] as consequences of long-term amphetamine and methamphetamine use. Together with our previous observation of impaired inhibitory control in khat users [33], this suggests that khat use may be associated with a general decrement in cognitive control. Another, not necessarily exclusive possibility is that the impairments on tasks measuring mental flexibility and WM were the result of transitory khat-induced withdrawal symptoms. Indeed, chronic khat users experience withdrawal symptoms during the first days, especially sleeping problems, depressive states, attentional problems, and intense cravings [5]. Moreover, given that cathine stays longer than 24 hours in the body, it cannot be excluded that our pattern of results is due to possible acute effects potentially masking or potentiating longer-term effects [5].

It is important to emphasize that the causal relation between cognitive impariment and the regular use of khat is not necessarily straightforward. For instance, we cannot exclude that pre-existing genetic or neurodevelopmental factors may play a mediating role. What we can exclude are contributions from other drugs, to which our khat users were barely exposed, and from individual characteristics as age or intelligence, which do impact performance on working-memory tasks [50]; [51]; [52]; [53] but were controlled in this study.

Although data on the density of DA receptors in khat users are not yet available, one may speculate that khat users suffer from the impaired functioning of dopaminergic receptors in the frontostriatal circuit. Indeed, the striatum is assumed to underlie the ability to flexibly alter cognitive representations [24] and to serve as a gate to modulate when to update information in the PFC structures subserving WM [25].

The present findings raise the question whether khat users also show impairments in other cognitive control functions, such as strategic planning and decision making [8]. The acute effect of khat on cognitive functions needs to be investigated as well. Moreover, it would very useful to explore the direct effect of khat use on the brain. It remains to be demonstrated, for instance, that khat use produces changes at the neuromodulatory (reduced functioning of DAD2 receptors) and functional level (dysfunction in PFC and striatum) that may be proportional to the degree of behavioural performance deficits. Of particular interest would be to investigate whether khat users suffer from impulsive behaviour—given that cathinone has been found to enhance aggressive behavior in isolated rats [54] and that dysfunctional impulsivity has been associated with genetic markers of striatal dopamine [34].

As pointed out, the observations that khat use is apparently associated with all three major functions of cognitive control (WM monitoring/updating, flexibility, and inhibition: [37]) suggest a broad and general impact of khat use on human cognition. Accordingly, using khat can be expected to affect a broad range of everyday behavior, ranging from car driving to work performance and social behavior.
